# Cofactors of wheat-dependent exercise-induced anaphylaxis do not increase highly individual gliadin absorption in healthy volunteers

**DOI:** 10.1186/s13601-019-0260-0

**Published:** 2019-03-25

**Authors:** Katharina Anne Scherf, Ann-Christin Lindenau, Luzia Valentini, Maria Carmen Collado, Izaskun García-Mantrana, Morten Christensen, Dirk Tomsitz, Claudia Kugler, Tilo Biedermann, Knut Brockow

**Affiliations:** 1 Leibniz-Institute for Food Systems Biology, Technical University of Munich, Lise-Meitner-Strasse 34, 85354 Freising, Germany; 2 Department of Agriculture and Food Sciences, Section of Dietetics, University of Applied Sciences Neubrandenburg, Brodaer Str. 2, 17033 Neubrandenburg, Germany; 3 Department of Biotechnology, Institute of Agrochemistry and Food Technology, Spanish National Research Council (IATA-CSIC), Av. Catedrático Agustín Escardino 7, 46980 Valencia, Spain; 4 Department of Dermatology and Allergy Centre, Odense Research Center for Anaphylaxis (ORCA), Odense University Hospital, 5000 Odense, Denmark; 5 Department of Dermatology and Allergy Biederstein, Technical University of Munich, Biedersteiner Strasse 29, 80802 Munich, Germany

**Keywords:** Cofactor, Gliadin, Gluten, Gut microbiota, Intestinal permeability, Wheat allergy, Wheat-dependent exercise induced anaphylaxis (WDEIA), Zonulin

## Abstract

**Background:**

In wheat-dependent exercise-induced anaphylaxis (WDEIA), cofactors such as exercise, acetylsalicylic acid (ASA), alcohol or unfavorable climatic conditions are required to elicit a reaction to wheat products. The mechanism of action of these cofactors is unknown, but an increase of gliadin absorption has been speculated. Our objectives were to study gliadin absorption with and without cofactors and to correlate plasma gliadin levels with factors influencing protein absorption in healthy volunteers.

**Methods:**

Twelve healthy probands (six males, six females; aged 20–56 years) ingested 32 g of gluten without any cofactor or in combination with cofactors aerobic and anaerobic exercise, ASA, alcohol and pantoprazole. Gliadin serum levels were measured up to 120 min afterwards and the intestinal barrier function protein zonulin in stool was collected before and after the procedure; both were measured by ELISA. Stool microbiota profile was obtained by 16S gene sequencing.

**Results:**

Within 15 min after gluten intake, gliadin concentrations in blood serum increased from baseline in all subjects reaching highly variable peak levels after 15–90 min. Addition of cofactors did not lead to substantially higher gliadin levels, although variability of levels was higher with differences between individuals (p < 0.001) and increased levels at later time points. Zonulin levels in stool were associated neither with addition of cofactors nor with peak gliadin concentrations. There were no differences in gut microbiota between the different interventions, although the composition of microbiota (p < 0.001) and the redundancy discriminant analysis (p < 0.007) differed in probands with low versus high stool zonulin levels.

**Conclusion:**

The adsorption of gliadin in the gut in healthy volunteers is less dependent on cofactors than has been hypothesized. Patients with WDEIA may have a predisposition needed for the additional effect of cofactors, e.g., hyperresponsive or damaged intestinal epithelium. Alternatively, other mechanisms, such as cofactor-induced blood flow redistribution, increased activity of tissue transglutaminase, or increases in plasma osmolality and acidosis inducing basophil and mast cell histamine release may play the major role in WDEIA.

## Background

IgE-mediated wheat allergies can be classified into respiratory allergy (baker’s asthma and rhinitis), wheat food allergy and wheat-dependent exercise-induced anaphylaxis (WDEIA) [1, 2]. WDEIA patients tolerate ingestion of wheat products alone, but experience a severe type I allergic reaction when wheat ingestion is combined with augmenting cofactors [3, 4]. The most prevalent cofactors are exercise (80%), alcohol (25%) and nonsteroidal anti-inflammatory drugs (NSAIDs, 9%), e.g., acetylsalicylic acid (ASA), but others such as infections, stress, menstruation and seasonal or unfavorable climatic conditions have been reported [5–7]. Typical symptoms of WDEIA are pruritus, angioedema and urticaria that appear within a few minutes of exercise and can progress to dyspnea, hypotension, vomiting and potentially life-threatening anaphylactic shock [8].

Data from different studies suggest that cofactors play a role in up to 30% of all anaphylactic reactions in adults [6]. However, the precise mode of action of cofactors has not been elucidated yet, not even in WDEIA, the best-studied model of cofactor-induced anaphylaxis [5, 9]. In general, different exercise-induced modes of action are possible: (1) decreased activation threshold of mast cells and basophils, (2) blood flow redistribution and transient plasma hyperosmolality [10] and (3) increased bioavailability of allergens through increased gastrointestinal permeability. Whereas the first option is neither likely nor supported by consistent data, the second option has been shown to increase histamine releasability. The third option has been supported by several publications: Intake of proton pump inhibitors (PPI) suppresses gastric acids and leads to less efficient degradation of otherwise labile allergens during digestion. These may reach the small intestine and cause de novo sensitization towards dietary proteins [11, 12]. Exercise is known to cause splanchnic hypoperfusion that may lead to damage of intestinal epithelial cells and subsequent increase of intestinal permeability [13, 14]. In lysozyme-sensitized mice, acute exercise strongly induced allergen leakage from the gastrointestinal tract into the circulation [15]. NSAIDs have also been reported to increase absorption of allergens in the small intestine [16] and cause mild small intestinal inflammation associated with increased intestinal permeability [17]. The mode of action involves inhibition of prostaglandin-endoperoxide synthase 1 [also known as cyclooxygenase (COX) 1] and COX2, as well as topical effects that disrupt membrane and mucus phospholipids and uncouple mitochondrial oxidative phosphorylation [17, 18]. Additionally, NSAIDs may directly affect mast cell degranulation [19]. Increased intestinal permeability may also be caused by alcohol mainly through oxidative stress [20]. Because patients with adverse food reactions had persistent alterations of intestinal permeability, this seems to be a key factor in allergy development [21]. Intestinal permeability is regulated by tight junctions, multiple protein complexes located at the apical ends of the lateral membranes of intestinal epithelial cells. Four integral transmembrane proteins (occludin, claudins, junctional adhesion molecule and tricellulin) regulate the paracellular passage of compounds and interact with the actin cytoskeleton through intracellular scaffold proteins (zonula occludens proteins and cingulin) [22]. Zonulin, a human intestinal homolog of zonula occludens toxin from *Vibrio cholerae*, is known to dysregulate tight junctions and increased zonulin expression has been observed, e.g., in celiac disease patients [23].

Furthermore, shifts in the gut microbiota profile have been associated with susceptibility to food allergy and may play a causal role [24]. Recently, a high relative abundance of *Clostridium* sensu stricto was associated with an increased risk of IgE-mediated food allergy in infants [25], whereas a distinct microbiota composition may exert protection from food allergies [26]. Our understanding of cause-effect relationships is only at the very beginning, but microbiota may have either direct immunologic effects or induce other mechanisms including control of intestinal barrier function [27]. For example, colonization with *Clostridia* species increased expression of barrier-promoting IL-22 and decreased intestinal permeability [28].

Taking all of the above together, regulation of intestinal permeability seems to be a major factor determining development, onset and severity of food allergenic reactions. Therefore, the main aims of our study were to assess the influence of cofactors for WDEIA on gliadin absorption in twelve healthy volunteers after gluten challenge and elucidate fundamental mechanisms involved in the regulation. Additional analyses of zonulin and microbiota in stool samples were carried out to clarify possible associations between gliadin absorption and intestinal permeability as well as composition of microbiota.

## Methods

### Probands

Twelve healthy volunteers (P1–P12, six females, six males, age 20–56 years, mean age 33 ± 11 years) participated in this prospective, explorative intervention study conducted at the Department of Dermatology and Allergy Biederstein from June to July 2015. Proband characteristics are shown in Table 1. Inclusion criteria were overall health and a minimum age of 18 years. Exclusion criteria were wheat allergy, pregnancy, cardiovascular diseases, gastrointestinal diseases, diabetes mellitus, and alcohol abuse. The study was approved by the Institutional Review Board of the Technical University of Munich (project no 2679/10) and informed consent was obtained from all participants.

**Table 1:** Characteristics of probands P1–P12

Proband	Sex	Age (years)	BMI (kg/m^2^)	Atopic diseases	Allergies
P1	Female	28	19	AR	Horse dander, grass pollen
P2	Male	20	21	AR	Unknown
P3	Male	23	25	AB	House dust mites
P4	Female	28	20		
P5	Male	27	22	AR	Birch pollen, house dust mites
P6	Male	30	24		
P7	Male	27	25	AR	Grass pollen, birch pollen, house dust mites
P8	Female	56	25	AR	Unknown
P9	Female	54	22		
P10	Female	44	28	AR	Unknown
P11	Female	31	20		
P12	Male	29	28	AR	Seafood

Sex, age, body mass index and atopy

*AB* bronchial asthma, *AR* allergic rhinitis, *BMI* body mass index

### Characterization of gluten used for oral challenge

Wheat gluten was obtained from Bösen Reform- und Mühlenbäckerei GmbH (Langenfeld, Germany). The crude protein content was determined by the Dumas combustion principle and the composition of gluten was analyzed by reserved-phase high-performance liquid chromatography calibrated with Prolamin Working Group-gliadin [29] as described in detail by Schalk et al. [30]. One gram of gluten had 718 mg crude protein, composed of 39 mg ω5-gliadins, 43 mg ω1,2-gliadins, 236 mg α-gliadins, 153 mg γ-gliadins, 61 mg high-molecular-weight glutenin subunits (HMW-GS) and 133 mg low-molecular-weight glutenin subunits (LMW-GS).

### Study design and challenge protocol

**Fig. 1:**
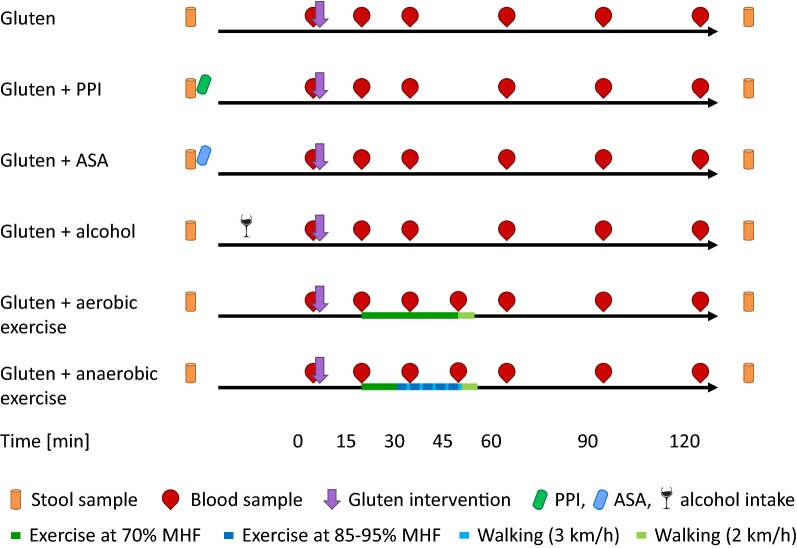
Study design and challenge protocol. *ASA* acetyl salicylic acid, *MHF* maximal heart frequency, *PPI* proton pump inhibitor

The six interventions (gluten, and gluten + cofactors) were carried out in randomized order with at least 48 h in between two interventions and at least 3 h fasting prior to challenge. The cofactors tested were PPI, ASA, alcohol, aerobic exercise and anaerobic exercise (Fig. 1). For gluten intervention without cofactors, 32 g of gluten was eaten as “bread” baked with 64 ml of water and 0.3 g of salt at 180 °C for 20 min [9]. For gluten intervention with PPI or ASA, 40 mg of Pantoprazol (Hexal, Holzkirchen, Germany) or 1000 mg of ASA (Bayer, Leverkusen, Germany) were administered 30 min prior to gluten intake. For gluten intervention with alcohol, the probands drank individual quantities of ethanol (95%, v/v, diluted with ice tea, 1:5, v/v) 15 min prior to gluten intake to reach a target blood alcohol level of 0.5 per mill considering sex and body weight. Aerobic and anaerobic exercise were both performed 15 min after gluten intake. For aerobic exercise, probands ran on a treadmill (HP Cosmos, Traunstein, Germany) at a pulse frequency of 65–75% of the maximal heart frequency (MHF, estimated by subtracting the proband’s age from 220) for 30 min. For anaerobic exercise, probands ran on a treadmill at a pulse frequency of 70% of MHF for 11 min, followed by five alternating rounds of running at a pulse frequency of 85–95% of MHF for 3 min and walking at a pace of 3 km/h for 1 min. Both exercise phases ended with a recovery phase of 5 min (walking at a pace of 2 km/h).

In total, seven stool samples were collected, one prior to the beginning of the study and the first stool sample after each intervention. Six blood samples were taken at 0 min (prior to gluten intake) and at 15 min, 30 min, 60 min, 90 min and 120 min after gluten intake for each intervention to monitor serum gliadin levels. An additional blood sample was taken 45 min into the interventions with aerobic and anaerobic exercise. In some probands, the direct effects of cofactors on the unspecific reactivity to histamine and codeine were analysed before and after the intervention, by the skin prick test to histamine 10 mg/ml and codeine phosphate 1% [for alcohol (n = 8) and anaerobic exercise (n = 5)] and by the basophil activation test (FlowCAST, Bühlmann, Schönenbuch, Schweiz) to both positive controls anti-IgE receptor antigen and *N*-formylmethionine-leucyl-phenylalanine [for alcohol (n = 8), ASS (n = 3) and anaerobic exercise (n = 7)].

### Serum gliadin concentrations

Blood samples were centrifuged (2500×*g*, 10 min, 20 °C) and serum was frozen at − 22 °C until analysis using the protocol described by Matsuo et al. [31] and Kohno et al. [32] with slight modifications. Serum (400 µl) was diluted with 80 µl ultrapure water and 1120 µl ethanol (95%, v/v), mixed for 1 min and heated to 95 °C for 10 min. The cooled samples were centrifuged (20,000×*g*, 30 min, 15 °C) and the supernatant transferred to a new tube. The residue was re-extracted with 300 µl of 60% ethanol (v/v), the mixture centrifuged again as described above and the combined supernatants were dried under reduced pressure in a vacuum centrifuge (16 h, 30 °C, 0.8 mPa). The dried extracts were reconstituted directly in 120 µl of sample diluent and measured using the Ridascreen^®^ Gliadin competitive ELISA kit (r-biopharm, Darmstadt, Germany) that detects intact gluten proteins and gluten peptides [33]. All ELISA analyses were performed in duplicates in a separate, closed room where the surfaces had been cleaned with 60% ethanol (v/v) to prevent contamination with gluten. The ELISA procedure was carried out according to the kit manual and the absorbances measured at 450 nm (Expert 96 microplate reader, Asys Hitech, Eugendorf, Austria).

### Zonulin concentrations in stool samples

Zonulin concentrations were assessed in stool as marker for intestinal permeability using the IDK^®^ Zonulin ELISA kit (Immundiagnostik AG, Bensheim, Germany).

### DNA extraction and 16S gene sequencing in stool samples

Total fecal DNA was isolated from 100 to 125 mg of feces using the MasterPure Complete DNA and RNA Purification Kit (Epicentre, Madison, USA) as previously described [34]. Isolated DNA concentrations were measured using a Qubit^®^ 2.0 Fluorometer (Life Technology, Carlsbad, CA, USA) and normalized to 5 ng/μL for 16S rDNA gene (V3–V4 region) amplification using Nextera XT Index Kit (Illumina, San Diego, USA). Amplicons were checked with a Bioanalyzer DNA 1000 chip and libraries were sequenced using a 2 × 300 pb paired-end run (MiSeq Reagent kit v3) on a MiSeq-Illumina platform (FISABIO sequencing service, Valencia, Spain). Controls during DNA extraction and polymerase chain reaction.

### Bioinformatics and statistical analysis

Data processing was performed using the QIIME pipeline (version 1.9.0, default parameters [34]). Chimeric sequences and sequences that could not be aligned were removed from the data set. An open reference operational taxonomic unit (OTU) picking method using 97% identity to the Greengenes 13_8 database was selected. OTUs present in < 0.01 and those classified as Cyanobacteria and Chloroplasts, were removed from the dataset. Alpha diversity indices (Chao1 and Shannon) and beta diversity using UNIFRAC (phylogenetic) and Bray–Curtis distance (non-phylogenetic) among samples and PERMANOVA were used to test significance. Calypso software (http://cgenome.net/calypso/) was used with total sum normalization (TSS) for the statistical analysis, and also, multivariate test. Linear discriminant analysis (LDA) effect size (LEfSe) algorithm was used to identify the most differentially abundant taxa according to conditions.

Areas under the curve of ELISA measurements (AUC) were calculated using OriginPro 8.5.1 (OriginLab Corporation, Northampton, MA, USA). Two-way analysis of variance (ANOVA) with proband and intervention as factors and Tukey’s post hoc test at p < 0.05 was used to identify significant differences between probands or interventions. Spearman correlations (significant at p < 0.05) were carried out between all possible combinations of zonulin levels, peak gliadin concentrations, AUC and relative abundances of bacterial groups. SigmaPlot 12.0 (Systat Software, San Jose, CA, USA) was used for ANOVA and correlation analyses.

## Results

Twelve healthy volunteers with (n = 8) and without (n = 4) atopic diseases years participated in the study (Table 1). None of the probands had atopic dermatitis, hives, drug allergies or ever experienced an allergic reaction after consumption of wheat-based foods, protein supplements or cosmetics containing hydrolyzed wheat proteins.

### Serum gliadin concentrations

**Fig. 2:**
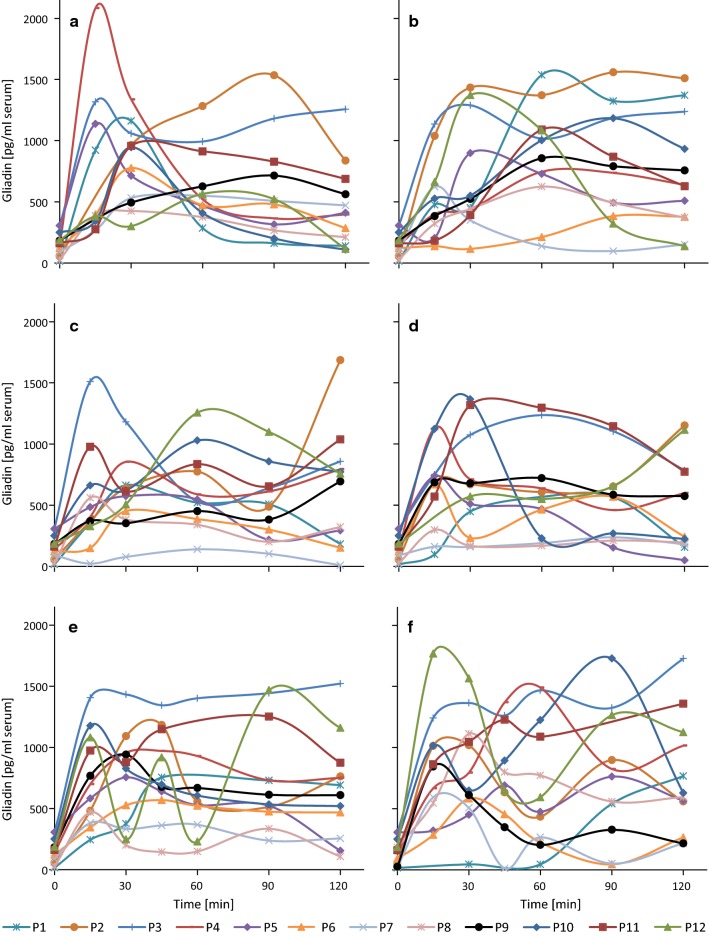
Gliadin concentrations in blood serum after different interventions. Gliadin (pg/ml blood serum) after intervention with 32 g of gluten without cofactor (**a**) or with 32 g of gluten and different cofactors, proton pump inhibitor (**b**), acetylsalicylic acid (**c**), alcohol (**d**), aerobic exercise (**e**) or anaerobic exercise (**f**) in probands 1–12 (P1–P12)

Within 15 min after gluten intake, gliadin concentrations in blood serum increased from baseline in all subjects, reaching peak levels between 15 and 90 min (Fig. 2a). Peak levels were highly individual ranging from 425 pg/ml (P8) to 2084 pg/ml (P4) (Table 2), as was the acceleration of the increase. After addition of the various cofactors (Fig. 2b–f), there were no obvious differences compared to gluten intervention without cofactors, although there was a higher variability of levels between 15 and 120 min and increases at later time points. The true maximal gliadin concentration cannot be reported for those curves where the peak levels occurred at 120 min (e.g., P2 and P9 with gluten + ASA, Fig. 2c), because this was the last time point analyzed and the maximum most likely occurred afterwards. The mean values over the twelve probands between the six interventions were not significantly different (two-way ANOVA with proband and intervention as factors and Tukey’s post hoc test, p = 0.208), so that no intervention generally led to higher or lower peak gliadin concentrations. However, there were individual differences (p < 0.001), because P6, P7 and P8 had significantly lower peak gliadin concentrations irrespective of the cofactor than P2, P3, P4, P11 and P12 (Table 2). Considering the six interventions per proband, the highest peak gliadin concentrations occurred with gluten + anaerobic exercise in 5 out of 12 probands (P3, P8, P9, P11 and P12), with gluten alone in 3 (P4–P6), with gluten + PPI in 2 (P1 and P7) and with gluten + ASA (P2) and gluten + alcohol (P10) in 1 proband each.

**Table 2:** Gliadin concentrations in serum at baseline and at the peak (pg/ml) after each intervention

Proband	Basal gliadin level	Gluten		Gluten + PPI		Gluten + ASA		Gluten + alcohol		Gluten + aerobic exercise		Gluten + anaerobic exercise		Mean (P) (n = 6)^3^
	(pg/ml)	(pg/ml)^1^	(min)^2^	(pg/ml)^1^	(min)^2^	(pg/ml)^1^	(min)^2^	(pg/ml)^1^	(min)^2^	(pg/ml)^1^	(min)^2^	(pg/ml)^1^	(min)^2^	(pg/ml)^1^
P1	15	1161	30	*1538*	60	664	30	568	60	754	45	767	120	909^ACD^
P2	57	1535	90	1559	90	*1686*	120	1153	120	1184	45	1019	30	1356^CD^
P3	306	1318	15	1289	30	1511	15	1237	60	1522	120	*1727*	120	1434^D^
P4	141	*2084*	15	737	90	855	30	1130	15	971	45	1491	60	1211^CD^
P5	306	*1137*	15	898	30	574	30	738	15	755	30	762	90	811^ABC^
P6	121	*777*	30	384	90	452	30	669	15	569	45	583	30	572^AB^
P7	90	549	60	*622*	15	139	60	234	90	382	15	594	15	420^A^
P8	26	425	30	625	60	560	15	298	15	469	15	*1113*	30	582^AB^
P9	184	715	90	857	60	694	120	722	60	942	30	*1729*	90	943^ABCD^
P10	250	946	30	1183	90	1029	60	*1366*	30	1177	15	844	15	1091^BCD^
P11	160	957	30	1091	60	1038	120	1319	30	1252	90	*1359*	120	1169^CD^
P12	186	565	60	1374	30	1257	60	1117	120	1468	90	*1767*	15	1258^CD^
Median	151	952		995		775		928		957		1066		
Mean (I)^4^	154	1014^A^		1013^A^		872^A^		879^A^		954^A^		1146^A^		
SEM	26	137		111		130		113		109		131		

Values in italics designate the highest peak gliadin concentration for each proband

*ASA* acetyl salicylic acid, *PPI* proton pump inhibitor, *SEM* standard error of the mean

^1^Peak gliadin concentration; ^2^time when peak gliadin concentration is reached; ^3^mean (P) designates the mean peak gliadin concentration of all six interventions per proband displayed per row. Different superscript capital letters indicate significant differences between means (P) of probands (two-way ANOVA, Tukey’s test, p < 0.001); ^4^mean (I) designates the mean peak gliadin concentration of all twelve probands per intervention displayed per column. There were no significant differences between means (I) (two-way ANOVA, Tukey’s test, p = 0.208), which is why all values are marked with the same superscript capital letter

The AUCs showed a similar overall picture compared to the peak gliadin concentrations, because the differences between the six interventions were not significant (p = 0.059), but inter-individual differences were apparent (p < 0.001) (Table 3). The AUCs of P6, P7 and P8 were lowest regardless of the cofactor, and those of P2, P3 and P11 were highest, with those of the remaining six probands in between. The overall AUC (n = 12 probands) was highest when gluten was combined with anaerobic exercise followed by gluten + PPI and gluten + aerobic exercise. The gluten + anaerobic exercise intervention led to the highest AUC values in 5 out of 12 probands (P4 and P9–P12), followed by the gluten + PPI intervention with the highest AUC values in 4 probands (P1, P2, P5 and P8) and the gluten intervention without cofactors with the highest AUC values in 2 probands (P6 and P7). P3 had the highest AUC with gluten + aerobic exercise. This pattern was similar for P1, P6, P9, P11 and P12, but somewhat different compared to that of peak gliadin concentrations for P2-P5, P7, P8 and P10 due to several reasons. P2 had steady high levels with gluten + PPI, but an exceptionally pronounced increase of the gliadin concentration at 120 min following the intervention with gluten + ASA (Fig. 2b, c). For P3, the curves looked very similar for gluten + aerobic and gluten + anaerobic exercise, but the concentration increased once more at 120 min with anaerobic exercise (Fig. 2e, f). For P4, P5, P8 and P10, high peak gliadin levels were reached with gluten alone (P4 and P5), with gluten + anaerobic exercise (P8) and with gluten + alcohol (P10), but with a fast increase and decrease, resulting in a lower AUC (Fig. 2).

**Table 3:** Gliadin levels in serum expressed as areas under the curve (AUC) after each intervention

Proband	Gluten	Gluten + PPI	Gluten + ASA	Gluten + alcohol	Gluten + aerobic exercise	Gluten + anaerobic exercise	Mean (P) (n = 6)^1^
	(area under the curve)						
P1	55,562	*123,910*	54,048	48,029	69,791	30,625	63,661^ABCD^
P2	12,6888	*158,897*	83,468	80,954	81,162	85,541	102,818^DEF^
P3	12,9994	132,946	99,449	119,755	*162,707*	158,978	133,971^F^
P4	94,900	71,054	74,315	75,912	94,554	*117,046*	87,964^CDE^
P5	65,023	*69,845*	49,792	44,235	61,865	66,233	59,499^ABC^
P6	*57,197*	29,083	36,223	50,788	55,538	44,863	45,615^AB^
P7	*55,415*	27,179	10,154	22,164	36,063	30,860	30,306^A^
P8	37,965	*54,002*	38,267	22,756	27,404	27,652	34,674^AB^
P9	66,551	79,698	50,321	74,795	79,739	*81,292*	72,066^BCDE^
P10	48,213	101,761	93,939	67,814	79,805	*129,075*	86,768^CDE^
P11	89,426	80,983	89,920	124,277	123,449	*129,796*	106,308^EF^
P12	48,217	86,500	99,754	72,879	101,717	*129,330*	89,733^CDE^
Median	61,110	80,340	64,182	70,346	79,772	83,416	
Mean (I)^2^	72,946^A^	84,655^A^	64,971^A^	67,030^A^	81,149^A^	85,941^A^	
SEM	8845	11,499	8438	9351	10,705	13,359	

Values in italics designate the highest AUC for each proband

*ASA* acetyl salicylic acid, *PPI* proton pump inhibitor, *SEM*, standard error of the mean

^1^Mean (P) designates the mean AUC of all six interventions per proband displayed per row. Different superscript capital letters indicate significant differences between means (P) of probands (two-way ANOVA, Tukey’s test, p < 0.001); ^2^Mean (I) designates the mean AUC of all twelve probands per intervention displayed per column. There were no significant differences between means (I) (two-way ANOVA, Tukey’s test, p = 0.059), which is why all values are marked with the same superscript capital letter

### Zonulin concentrations in stool samples

Next, the influence of gluten intervention with and without cofactors on zonulin levels was assessed in stool samples taken before (basal zonulin level) and after each intervention (Table 4). There were individual differences in basal zonulin levels ranging from 0 ng/ml (not detectable, P7) to 47.5 ng/ml (P3). Interventions caused an increase in zonulin levels of up to 1092% (P4, gluten + ASA) relative to baseline in all probands except P5, who had lower zonulin levels. Out of the six interventions, the highest individual zonulin levels were observed in 4 out of 12 probands (P4, P5, P7 and P11) after intervention with gluten + ASA, in 3 probands (P2, P3 and P6) with gluten + alcohol, in 2 probands each with gluten + aerobic exercise (P8 and P10) and with gluten + anaerobic exercise (P1 and P9) and in 1 proband with either gluten or gluten + PPI. Overall, the addition of cofactors was not associated with higher zonulin levels in stool (p = 0.400) compared to intervention with gluten alone. Zonulin levels were also not correlated to peak gliadin concentrations or AUC (Spearman correlation, p > 0.05). Additionally, cofactors neither led to an increase in the diameter of the skin prick tests to histamine or codeine phosphate, nor to elevated basophil activation to any cofactor tested (data not shown).

**Table 4:** Zonulin levels in stool at baseline (ng/ml) and after each intervention

Pro-band	Basal zonulin level	Gluten	Gluten + PPI	Gluten + ASA	Gluten + alcohol	Gluten + aerobic exercise	Gluten + anaerobic exercise	Mean (P) (n = 6)
	(ng/ml)							
P1	16.5^low^	44.5	16	10	16.5	45	82.5	35.8
P2	18^low^	89	26	39.5	108	38	6.5	51.2
P3	47.5^high^	8.5	21	16.5	78.5	n.d.	20.5	29.0
P4	12.5^low^	19.5	32	136.5	49	36	63	56.0
P5	27.5^high^	11	8	22	6	0	n.d.	9.4
P6	27^high^	33	27	n.d.	48.5	21.5	21	30.2
P7	0^low^	9.5	11	41	24	19	24	21.4
P8	32^high^	38	34	35	36.5	68	18.5	38.3
P9	25^low^	31.5	32	42	20.5	31.5	79	39.4
P10	33^high^	35.5	17.5	26.5	17	47.5	15	26.5
P11	25^low^	9.5	26.5	99.5	62.5	16.5	48.5	43.8
P12	29.5^high^	31.5	31.5	24	13	29.5	17.5	24.5
Median	26.0	31.5	26.3	35.0	30.3	31.5	21.0	
Mean (I)	24.5	30.1	23.5	44.8	40.0	32.0	36.0	
SEM	3.4	6.5	2.5	11.1	8.9	5.2	7.9	

*ASA* acetyl salicylic acid, *n.d.* not determined, *PPI* proton pump inhibitor, *SEM* standard error of the mean

Means (I) between six interventions (n = 12 probands) were not significantly different (p = 0.400, two-way ANOVA, Tukey’s test, with proband and intervention as factors), and neither were means (P) between probands (n = 6 interventions, p = 0.138)

### Microbiota in stool samples

**Fig. 3:**
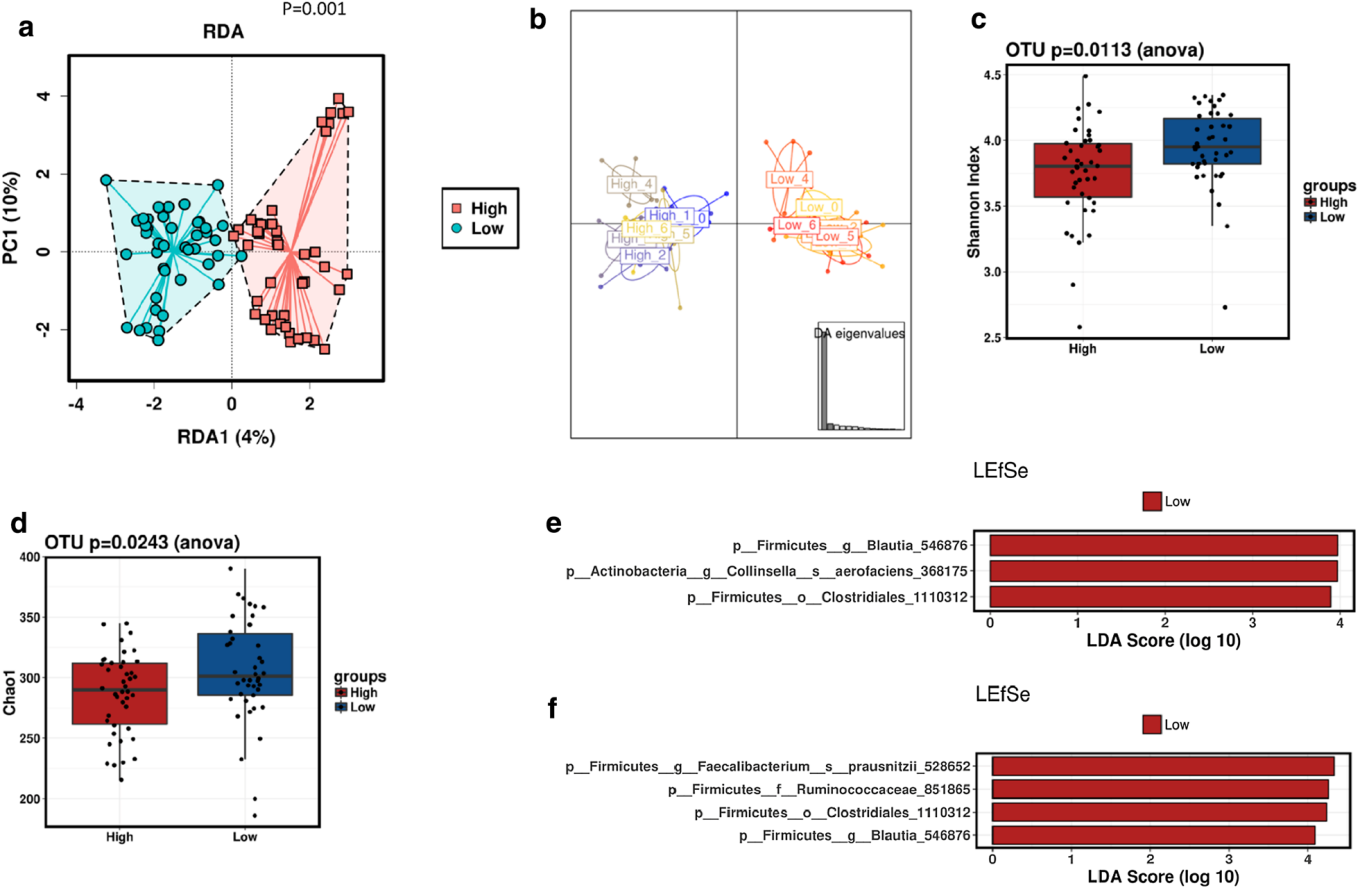
Microbiota composition and diversity after interventions and according to stool zonulin levels. Redundancy discriminant analysis (RDA) to divide probands into groups according to high or low zonulin group and intervention over all samples (**a**), Discriminant analysis of principal components (DAPC) plot (**b**), alpha diversity according to high or low zonulin group and intervention over all samples, Shannon index (**c**) and Chao1 (**d**), linear discriminant analysis (LDA) combined with effect size measurements (LeFSe) revealed specific bacterial groups at OTU level related to the low zonulin level group at baseline (**e**) and after gluten intervention (**f**). 0, baseline, 1, after intervention with gluten, 2–6, after intervention with gluten and different cofactors, 2, proton pump inhibitor, 3, acetyl salicylic acid, 4, alcohol, 5, aerobic exercise, 6, anaerobic exercise, *OTU* operational taxonomic unit

No differences in microbiota were found between the different interventions as compared to baseline (p = 0.775, Shannon index). Then, probands were stratified according to their zonulin stool levels at baseline into two groups representing low (average = 16.2 ± 8.5 ng/ml) and high (average = 32.8 ± 7.0 ng/ml) levels (p = 0.007). Multivariate RDA showed statistically significant differences in microbial composition between probands with higher and lower zonulin levels (p = 0.001) at baseline (Fig. 3a) regardless of the intervention (Fig. 3b).

In terms of alpha-diversity, lower bacterial diversity (p = 0.011, Shannon index) and richness (p = 0.024, Chao1 index) were observed at baseline in the higher zonulin level group compared to the lower zonulin level group (Fig. 3c, d). Moreover, we also observed significant negative correlations between zonulin in faeces and relative abundances of several bacterial groups, such as *Collinsella aerofaciens* (p = 0.003), *Bifidobacterium adolescentis* (p = 0.040) and unclassified species of *Coprococcus* (p = 0.028) and *Dorea* (p = 0.051), indicating that higher levels of these bacterial groups were related to lower levels of zonulin in faeces at baseline. Additionally, LEfSe analysis showed an enrichment of specific gut bacteria, such as *Collinsella aerofaciens*, and specific OTUs belonging to *Blautia* (OTU 546876) and *Clostridiales* (OTU 1110312) in the low zonulin level group (Fig. 3e).

After gluten intervention, LEfSe analysis showed that *Faecalibacterium prausnizii, Ruminococcacceae (OTU851865), Clostridiales (OTU 1110312)* and *Blautia (OTU 546876)* were related to the low level zonulin group (Fig. 3f). Furthermore, no significant associations between gut microbiota and serum gliadin concentrations were identified.

## Discussion

The mode of action of cofactors to induce and amplify anaphylaxis is still unknown [5, 35]. In WDEIA, the most prevalent hypothesis is that cofactors increase gliadin absorption leading to higher allergen levels [9, 32], possibly under the influence of zonulin as important regulator of intestinal permeability [36] and of microbial composition [37]. Exercise as cofactor alone is not sufficient to cause anaphylaxis, but it is likely that exercise combined with the ingestion of substances that damage the intestinal mucosa, such as ASA or alcohol, increase the risk of developing anaphylaxis. Other pathophysiological mechanisms discussed in WDEIA are increased activity of tissue transglutaminase in the gut mucosa, exercise-induced blood flow redistribution and mast cell heterogeneity, exercise-induced increases in plasma osmolality inducing basophil histamine release and exercise-induced acidosis and mast cell degranulation. Although these proposed mechanisms all appear to be valid, there is currently little to no experimental evidence to support either mode of action and an urgent need for further studies involving a global research network was identified [38].

The oral food challenge was performed using wheat gluten that contained all components (ω5-, ω1,2-, α- and γ-gliadins, HMW-GS and LMW-GS) that have been reported as allergens in WDEIA. Although ω5-gliadins are the most well-documented trigger for WDEIA, wheat-allergic individuals have also been reported to be sensitized to the other components [39] and thus wheat gluten appears to be most suitable to study WDEIA mechanisms.

This study shows that cofactors did not fundamentally raise serum peak gliadin levels or AUC in healthy volunteers, even though gliadin levels were somewhat more undulating and the mean and median AUC was highest for gluten + anaerobic exercise. We have previously shown that all patients with WDEIA and positive oral challenge to gluten had reactions at the time near the peak of serum gliadin levels [9]. However, clinical reaction levels were also highly individual in that study. Cofactors such as exercise or ASA did not always increase gliadin levels in a patient, whereas there was a general trend for higher gliadin levels, when higher gluten doses were combined with cofactors. Here, we found neither increased unspecific mast cell reactivity measured by skin prick tests nor for basophil reactivity in the blood. Further, cofactor addition and resulting gliadin levels neither correlated with fecal zonulin levels nor with the microbiota composition.

Short chain fatty acids are the products of bacterial fermentation of undigested carbohydrates in the intestine. Of them, butyrate improves the intestinal barrier by facilitating the assembly of tight junctions [40–42]. Moreover, some species of *Lactobacillus* and *Bifidobacterium*, also improve barrier function [43, 44]. Although all probands were healthy individuals and no significant differences in microbiota between interventions were seen, we observed some interindividual differences by sorting the probands according to the presence of high versus low levels of zonulin in feces. In our study we showed an enrichment of some butyrate producers such as *Faecalibacterium prausnizii*, *Blautia* and members of the *Ruminococcaceae* family in the lower zonulin level group. Negative correlations were also found between unclassified species of *Coprococcus* and *Dorea* and zonulin levels. Loss of microbial diversity appears as a constant finding of intestinal dysbiosis and is associated with several disorders [45, 46]. Changes in gut microbiota composition have been shown to affect intestinal barrier function by triggering intestinal zonulin release [36]. The lower zonulin level group also presented higher microbial diversity. This may indicate a better gut barrier integrity possibly due to the protective effect of a higher presence of beneficial bacteria. Harboring an adequate microbial composition may help maintain intestinal barrier integrity by down-regulating zonulin release in the gut. Changes in gut microbiota may be involved in increasing intestinal permeability in patients with WDEIA enabling cofactors to further increase absorption in the gut.

The main limitation of this exploratory study was to analyze gliadin absorption following gluten and cofactor challenge only in healthy volunteers, which may not have a predisposition to develop WDEIA. Susceptible individuals are likely to have a certain predisposition that promotes acute exercise-associated reactions and, therefore, further investigations in WDEIA patients and healthy controls are currently underway.

## Conclusion

In healthy volunteers, microbial colonization was significantly associated with stool zonulin, which regulates the function of tight junctions. However, zonulin was neither associated with total gliadin levels, nor were total gliadin levels significantly increased after the addition of cofactors. Gliadin levels following intake of gluten in healthy individuals were highly variable. Thus, in the mechanism of WDEIA, either the adsorption of gliadin in the gut is less dependent on cofactors than has been hypothesized, or patients with WDEIA have a predisposition for the action of cofactors (e.g., by mutations affecting zonulin) which is different from the situation in healthy volunteers.

## Abbreviations

ANOVA: analysis of variance
ASA: acetylsalicylic acid
AUC: area under the curve
COX: cyclooxygenase
DAPC: discriminant analysis of principal components
ELISA: enzyme-linked immunosorbent assay
HMW-GS: high-molecular-weight glutenin subunits
LEfSe: linear discriminant analysis effect size
LMW-GS: low-molecular-weight glutenin subunits
MHF: maximal heart frequency
NSAID: nonsteroidal anti-inflammatory drug
OTU: operational taxonomic unit
PCR: polymerase chain reaction
PPI: proton pump inhibitor
RDA: redundancy discriminant analysis
WDEIA: wheat-dependent exercise-induced anaphylaxis

## References

[1] Quirce S, Boyano-Martínez T, Díaz-Perales A (2016). Clinical presentation, allergens, and management of wheat allergy. Expert Rev Clin Immunol.

[2] Christensen MJ, Eller E, Mortz CG, Bindslev-Jensen C (2014). Patterns of suspected wheat-related allergy: a retrospective single-centre case note review in 156 patients. Clin Transl Allergy.

[3] Palosuo K, Alenius H, Varjonen E, Koivuluhta M, Mikkola J, Keskinen H (1999). A novel wheat gliadin as a cause of exercise-induced anaphylaxis. J Allergy Clin Immunol.

[4] Scherf KA, Brockow K, Biedermann T, Koehler P, Wieser H (2016). Wheat-dependent exercise-induced anaphylaxis. Clin Exp Allergy.

[5] Kennard L, Thomas I, Rukowski K, Azzu V, Yong PFK, Kasternow B (2018). A multicenter evaluation of diagnosis and management of omega-5 gliadin allergy (also known as wheat-dependent exercise-induced anaphylaxis) in 132 adults. J Allergy Clin Immunol.

[6] Wölbing F, Fischer J, Köberle M, Kaesler S, Biedermann T (2013). About the role and underlying mechanisms of cofactors in anaphylaxis. Allergy.

[7] Romano A, Scala E, Rumi G, Gaeta F, Caruso C, Alonzi C (2012). Lipid transfer proteins: the most frequent sensitizer in Italian subjects with food-dependent exercise-induced anaphylaxis. Clin Exp Allergy.

[8] Lehto M, Palosuo K, Varjonen E, Majuri ML, Andersson U, Reunala T, Alenius H (2003). Humoral and cellular responses to gliadin in wheat-dependent, exercise-induced anaphylaxis. Clin Exp Allergy.

[9] Brockow K, Kneissl D, Valentini L, Zelger O, Grosber M, Kugler C (2015). Using a gluten oral food challenge protocol to improve diagnosis of wheat-dependent exercise-induced anaphylaxis. J Allergy Clin Immunol.

[10] Barg W, Wolanczyk-Medrala A, Obojski A, Wytrychowski K, Panaszek B, Medrala W (2008). Food-dependent exercise-induced anaphylaxis: possible impact of increased basophil histamine releasability in hyperosmolar conditions. J Investig Allergol Clin Immunol.

[11] Untersmayr E, Jensen-Jarolim E (2006). The effect of gastric digestion on food allergy. Curr Opin Allergy Clin Immunol.

[12] Untersmayr E, Jensen-Jarolim E (2008). The role of protein digestibility and antacids on food allergy outcomes. J Allergy Clin Immunol.

[13] Pals KL, Chang RT, Ryan AJ, Gisolfi CV (1997). Effect of running intensity on intestinal permeability. J Appl Physiol.

[14] Karhu E, Forsgård RA, Alanko L, Alfthan H, Pussinen P, Hämäläinen E, Korpela R (2017). Exercise and gastrointestinal symptoms: running-induced changes in intestinal permeability and markers of gastrointestinal function in asymptomatic and symptomatic runners. Eur J Appl Physiol.

[15] Yano H, Kato Y, Matsuda T (2002). Acute exercise induces gastrointestinal leakage of allergen in lysozyme-sensitized mice. Eur J Appl Physiol.

[16] Matsuo H, Morimoto K, Akaki T, Kaneko S, Kusatake K, Kuroda T (2005). Exercise and aspirin increase levels of circulating gliadin peptides in patients with wheat-dependent exercise-induced anaphylaxis. Clin Exp Allergy.

[17] Sigthorsson G, Tibble J, Hayllar J, Menzies I, Macpherson A, Moots R (1998). Intestinal permeability and inflammation in patients on NSAIDs. Gut.

[18] Bjarnason I, Scarpignato C, Holmgren E, Olszewski M, Rainsford KD, Lanas A (2018). Mechanisms of damage to the gastrointestinal tract from nonsteroidal anti-inflammatory drugs. Gastroenterology.

[19] Suzuki Y, Ra C (2009). Analysis of the mechanism for the development of allergic skin inflammation and the application for its treatment: aspirin modulation of IgE-dependent mast cell activation: role of aspirin-induced exacerbation of immediate allergy. J Pharmacol Sci.

[20] Ferrier L, Berard F, Debrauwer L, Chabo C, Langella P, Bueno L, Fioramonti J (2006). Impairment of the intestinal barrier by ethanol involves enteric microflora and mast cell activation in rodents. Am J Pathol.

[21] Ventura MT, Polimeno L, Amoruso AC, Gatti F, Annoscia E, Marinaro M (2006). Intestinal permeability in patients with adverse reactions to food. Dig Liver Dis.

[22] Suzuki T (2013). Regulation of intestinal epithelial permeability by tight junctions. Cell Mol Life Sci.

[23] Lammers KM, Lu R, Brownley J, Lu B, Gerard C, Thomas K (2008). Gliadin induces an increase in intestinal permeability and zonulin release by binding to the chemokine receptor CXCR3. Gastroenterology.

[24] Savage JH, Lee-Sarwar KA, Sordillo J, Bunyavanich S, Zhou Y, O’Connor G (2018). A prospective microbiome-wide association study of food sensitization and food allergy in early childhood. Allergy.

[25] Ling Z, Li Z, Liu X, Cheng Y, Luo Y, Tong X (2014). Altered fecal microbiota composition associated with food allergy in infants. Appl Environ Microbiol.

[26] Diesner SC, Bergmayr C, Pfitzner B, Assmann V, Krishnamurthy D, Starkl P (2016). A distinct microbiota composition is associated with protection from food allergy in an oral mouse immunization model. Clin Immunol.

[27] Blázquez AB, Berin MC (2017). Microbiome and food allergy. Transl Res.

[28] Stefka AT, Feehley T, Tripathi P, Qiu J, McCoy K, Mazmanian SK (2014). Commensal bacteria protect against food allergen sensitization. Proc Natl Acad Sci USA.

[29] Van Eckert R, Berghofer E, Ciclitira PJ, Chirdo F, Denery-Papini S, Ellis H-J (2006). Towards a new gliadin reference material—isolation and characterisation. J Cereal Sci.

[30] Schalk K, Lexhaller B, Koehler P, Scherf KA (2017). Isolation and characterization of gluten protein types from wheat, rye, barley and oats for use as reference materials. PLoS ONE.

[31] Matsuo H, Dahlström J, Tanaka A, Kohno K, Takahashi H, Furumura M, Morita E (2008). Sensitivity and specificity of recombinant omega-5 gliadin-specific IgE measurement for the diagnosis of wheat-dependent exercise-induced anaphylaxis. Allergy.

[32] Kohno K, Matsuo H, Takahashi H, Niihara H, Chinuki Y, Kaneko S (2013). Serum gliadin monitoring extracts patients with false negative results in challenge tests for the diagnosis of wheat-dependent exercise-induced anaphylaxis. Allergo Int.

[33] Scherf KA, Poms RE (2016). Recent advances in analytical methods for tracing gluten. J Cereal Sci.

[34] Boix-Amoros A, Collado MC, Mira A (2016). Relationship between milk microbiota, bacterial load, macronutrients, and human cells during lactation. Front Microbiol.

[35] Christensen MJ, Eller E, Mortz CG, Brockow K, Bindslev-Jensen C (2018). Exercise lowers threshold and increases severity, but wheat-dependent, exercise-induced anaphylaxis can be elicited at rest. J Allergy Clin Immunol Pract.

[36] Fasano A (2012). Intestinal permeability and its regulation by zonulin: diagnostic and therapeutic implications. Clin Gastroenterol Hepatol.

[37] Thursby E, Juge N (2017). Introduction to the human gut microbiota. Biochem J.

[38] Ansley L, Bonini M, Delgado L, Del Giacco S, Du Toit G, Khaitov M (2015). Pathophysiological mechanisms of exercise-induced anaphylaxis: an EAACI position statement. Allergy.

[39] Borres MP, Maruyama N, Sato S, Ebisawa M (2016). Recent advances in component resolved diagnosis in food allergy. Allergol Int.

[40] Wong JMW, de Souza R, Kendall CWC, Emam A, Jenkins DJA (2006). Colonic health: fermentation and short chain fatty acids. J Clin Gastroenterol.

[41] Peng L, Li Z-R, Green RS, Holzman IR, Lin J (2009). Butyrate enhances the intestinal barrier by facilitating tight junction assembly via activation of AMP-activated protein kinase in Caco-2 cell monolayers. J Nutr.

[42] Geirnaert A, Calatayud M, Grootaert C, Laukens D, Devriese S, Smagghe G (2017). Butyrate-producing bacteria supplemented in vitro to Crohn’s disease patient microbiota increased butyrate production and enhanced intestinal epithelial barrier integrity. Sci Rep.

[43] Anderson RC, Cookson AL, McNabb WC, Park Z, McCann MJ, Kelly WJ (2010). *Lactobacillus plantarum* MB452 enhances the function of the intestinal barrier by increasing the expression levels of genes involved in tight junction formation. BMC Microbiol.

[44] Sultana R, McBain AJ, O’Neill CA (2013). Strain-dependent augmentation of tight-junction barrier function in human primary epidermal keratinocytes by Lactobacillus and Bifidobacterium lysates. Appl Environ Microbiol.

[45] Human Microbiome Project Consortium THMP (2012). Structure, function and diversity of the healthy human microbiome. Nature.

[46] Menni C, Jackson MA, Pallister T, Steves CJ, Spector TD, Valdes AM (2017). Gut microbiome diversity and high-fibre intake are related to lower long-term weight gain. Int J Obes.

